# A Novel Vector for Construction of Markerless Multicopy Overexpression Transformants in *Pichia pastoris*

**DOI:** 10.3389/fmicb.2017.01698

**Published:** 2017-09-11

**Authors:** Ding Li, Bo Zhang, Shuting Li, Jie Zhou, Hui Cao, Yan Huang, Zhongli Cui

**Affiliations:** ^1^Key Laboratory of Agricultural Environmental Microbiology, College of Life Sciences, Nanjing Agricultural University Nanjing, China; ^2^College of Life Sciences, Nanjing Agricultural University Nanjing, China

**Keywords:** markerless genetic manipulation, novel expression vector, dosage effect, phytase appA, *Pichia pastoris* expression system

## Abstract

*Pichia pastoris* is widely used as a platform for heterologous protein expression because of its high volumetric productivity. Multicopy integration of the target gene is commonly used to improve the production of the target protein. Cre/*lox* recombination system is a powerful tool for the marker rescue during multiple integrations with one selection marker. Here we reported a novel expression vector based on the Cre/*lox* recombination system for multiple integrations of target gene to construct multicopy expression strain of *P. pastoris*. P_AOX1_ promoter was fused to *cre* to construct a methanol inducible Cre recombinase. The leakage expression of Cre recombinase in *Escherichia coli* was blocked by introducing the operator gene *lacO*. The expression vector designed pMCO-AOXα was stable in *E. coli* and could effectively rescue the Zeocin resistance gene for next round of integration in *P. pastoris*. Phytase AppA from *E. coli* was chosen as a reporter gene. Transformants with 2–16 copies of *appA* were constructed by using a single antibiotic. Expression of *appA* was gene dosage dependent when <12 copies were integrated. The protein yield increased 4.45-folds when 12 copies of *appA* were integrated comparing with the single copy integration. Our results showed that pMCO-AOXα was highly effective for rational construction of multicopy transformat in *P. pastoris*.

## Introduction

Over the past few decades, *Pichia pastoris* has developed into a highly popular expression host for production of recombinant protein (Spohner et al., 2015). Several approaches have been designed to improve the expression level of the target protein, such as codon optimization (Mellitzer et al., 2012; Yu et al., 2013), modification of a signal peptide (Liu et al., 2005; Li et al., 2015), selection of promoters with strong transcription strength (Çalık et al., 2015; Parashar and Satyanarayana, 2016), increasing the gene copy number and co-expression helper protein factors (Zhu et al., 2009; Yang et al., 2016). Multicopy integration of the target genes into the genome of *P. pastoris* was considered to be the most efficient strategy. To integrate multiple copies of a foreign gene, four methods have been employed: repeat transformations with the target gene, *in vitro* multimerization, direct selection using high concentrations of antibiotic and the post-transformational vector amplification (PTVA) method (Aw and Polizzi, 2013). The most frequently employed method is to directly select the transformation mixture with increasing concentrations of antibiotics. Among the transformants, some clones may be multicopy transformants (Lincereghino and Lincereghino, 2007). However, these methods should operate with antibiotics at high concentration or with multiple selection markers. The optional selection markers offered by expression hosts are limited. If they are are fully utilized, the extra selection markers required by subsequent integrations may be hardly investigated. The limited resources of selection markers that can be used in *P. pastoris* is the main bottleneck that hinders the arbitrary integration of the target gene or genes encoding helper protein factors into the *P. pastoris* genome.

Markerless manipulation in the host is desirable for multiple gene integrations or deletions (Leibig et al., 2008; Weng et al., 2009; Tuntufye and Goddeeris, 2011). Bacteriophage P1 Cre recombinase has proved to be a powerful tool for the removal of selection markers (Sauer, 1987). The Cre/*lox* recombination system has been used in a wide variety of eukaryotes, including yeasts (Gueldener et al., 2002). However, it has not yet been used to remove selection markers in the construction of high-copy transformants for protein expression in *P. pastoris*. Generally, markerless gene deletion in yeast is carried out in three steps. First, a selection marker flanked by *loxP* sites and recombinant arms was integrated into the target gene; then, a vector containing an inductive *cre* gene was introduced and induced to excise the marker gene from *loxP* sites; finally, the selection marker was eliminated for next cycle of manipulation. In this method, the *cre* gene and the disruption cassette cannot be combined in one vector because of the instability caused by possible leakage expression of Cre recombinase; the procedure is tedious and time-consuming. To overcome this disadvantage, a PCR fusion method which combined the *cre* gene and the disruption cassette in *P. pastoris* was presented in 2011 (Pan et al., 2011). Meanwhile, by introducing an intron in the *cre* gene, Agaphonov and Alexandrov (2014) constructed a single vector containing the *cre* gene and the disruption cassette. Both methods have proved to be effective in yeast genome editing.

Multicopy transformant screening with high concentrations of antibiotics is random. In this study, a vector containing the *cre* gene and the selection marker excision cassette was developed for construction of a *P. pastoris* expression strain with a designed copy number of heterologous protein genes.

## Materials and methods

### Strains, plasmids, and culture medium

The subcloning and construction of recombinant plasmids was carried out in *E. coli* DH10B (Invitrogen). The *P. pastoris* GS115 strain (Invitrogen) was used as a host for the expression of heterogenous proteins. The vectors pPIC3.5K and pPICZαA were purchased from Invitrogen. The vector pTSC (Yan et al., 2008) was stocked in this lab. pUC57-MCS7 (the original plasmid used for construction of recombinant expression vectors) was from GeneCreate Biological Engineering Co., Ltd, Wuhan, China. *E. coli* DH10B was cultured in Luria-Bertani medium [1% (w/v) tryptone, 0.5% (w/v) yeast extract, and 1% (w/v) NaCl, pH 7.0] at 37°C. The *P. pastoris* yeast strain was cultured in YPD medium [1% (w/v) yeast extract, 2% (w/v) peptone, 2% (w/v) dextrose]. YPDZ plates containing Zeocin (100 μg/mL) were used for the selection of positive *P. pastoris* transformants. Buffered minimal glycerol-complex medium (BMGY) was prepared with 2% (w/v) peptone, 1% (w/v) yeast extract, 1% (w/v) glycerol, 1.34% (w/v) yeast nitrogen base with ammonium sulfate but without amino acids, and 4 × 10^−5^% (w/v) biotin in 100 mM potassium phosphate buffer. The phosphate buffer was adjusted to pH 6.0. Buffered minimal methanol-complex medium (BMMY) was the same as BMGY, except that 0.5% (w/v) methanol was replaced with glycerol.

### Construction of the expression vector pMC

All primers used in the construction of the recombinant plasmid pMC are shown in Table 1; cleavage sites of restriction endonucleases are underlined. The schematic map of the construction of expression vector pMC is shown in Figure S1 (online resource) and can be briefly described as follows. A fragment with 7 multiple cloning sites was synthesized *in vitro* and cloned into pUC57-Kan-MCS-free, resulting in a pUC57-MCS7 plasmid. The *HIS4* gene was amplified by PCR from pPIC3.5K using the primer pairs HIS4-A-F/R and HIS4-B-F/R and was integrated into the pUC57-MCS7 plasmid. The *Xba* I on *HIS4* was synonymously mutated by overlap extension PCR. The fragment AOX1TT-P_TEF1_-P_EM7_-Zeocin^R^-CYC1TT-*lox66* was amplified from pPICZαA using the primer pairs ATEZC-F/R and was integrated into the pUC57-MCS7 plasmid. The *cre* gene was amplified from pTSC using the primer pairs Cre-F/R and was integrated into the pUC57-MCS7 plasmid. The fragment *lox71*-P_AOX1_ was amplified from pPICZαA using the primer pairs AOX-F/R and was integrated into the pUC57-MCS7 plasmid. Finally, the recombinant plasmid was named pMC.

**Table 1:** The primers used for construction of the expression vector.

**Primers**	**Sequence (5′–3′)**	**PCR product**
HIS4-A-F	GC**TCTAGA**ATGACATTTCCCTTGCTACCTG	*Xba* I-*HIS4*-*Bgl* II
HIS4-A-R	CCTTAACAGCATTGCGGTGAGCATCaAGACCTTCAACAGCAGCCAGATC	
HIS4-B-F	GATCTGGCTGCTGTTGAAGGTCTtGATGCTCACCGCAATGCTGTTAAGG	
HIS4-B-R	GA**AGATCT**TTAAATAAGTCCCAGTTTCTCCATAC	
ATEZC-F	ATAAGAAT**GCGGCCGC**GCCTTAGACATGACTGTTCCTCAG	*Not* I-AOX1TT-P_TEF1_-P_EM7_-Zeocin^R^-CYC1TT-*lox66*-*Spe* I
ATEZC-R	GG**ACTAGT**TACCGTTCGTATAATGTATGCTATACGAAGTTATCAGCTTGCAAATTAAAGCCTTCG	
Cre-F	CCG**CTCGAG**ATGTCCAATTTACTGACCGTACAC	*Xho* I-*Cre*-*Not* I
Cre-R	ATAAGAAT**GCGGCCGC**CTAATCGCCATCTTCCAGCAG	
AOX-F	ATAA**GGGCCC**TACCGTTCGTATAGCATACATTATACGAAGTTATGATCTAACATCCAAAGACGA	*Apa* I-*lox71*-P_AOX1_-*Xho* I
AOX-R	CCG**CTCGAG**CGTTTCGAATAATTAGTTGT	
AOX-R-uORF	CCG**CTCGAG**TCATTATCTTCTTCTTCTTCTTCTTCTTCTCATCGTTTCGAATAATTAGTTGT	*Apa* I-P_AOX1_-uORF-*Xho* I
AOX-R-*lacO*	CCG**CTCGAG**ggaattgttatccgctcacaattccCGTTTCGAATAATTAGTTGT	*Apa* I-P_AOX1_-*lacO*-*Xho* I
d*Xho* I-R	TACGGTCAGTAAATTGGACATggaattgttatccgctcacaatt	Deletion *Xho* I site
d*Xho* I-F	aattgtgagcggataacaattccATGTCCAATTTACTGACCGTA	
d*Not* I-R	GCGCCTGCTGGAAGATGGCGATTAGGCCTTAGACATGACTGTTCCTCAG	Deletion *Not* I site
d*Not* I-F	CTGAGGAACAGTCATGTCTAAGGCCTAATCGCCATCTTCCAGCAGGCGC	
d*Nco* I-R	AACGGCACTGGTCAACTTGGCCATGTTTAGTTCCTCACCTTGTCGTA	Deletion *Nco* I site
d*Nco* I-F	TACGACAAGGTGAGGAACTAAACATGGCCAAGTTGACCAGTGCCGT	
TT-F	AA**CTGCAG**ATAAGAATGCGGCCGCGCCTTAGACATGACTGTTCCTC	*Pst* I-*Not* I-AOX1TT-*Xba* I
TT-R	GC**TCTAGA**TCTCACTTAATCTTCTGTACTCTG	
P_AOX1_-F	AA**ACTAGT**GATCTAACATCCAAAGACGAAAGG	*Spe* I-P_AOX1_-αFactor-*EcoR* I-*Pst* I
αf-R	AA**CTGCAG**AGGAATTCAGCTTCAGCCTCTCTTTTCTCGAGAG	

### Identification of leakage expression of cre regulated by the AOX1 promoter in *E. coli*

Overnight cultures of *E. coli* DH10B (pMC) were harvested for extraction of total RNA. Total RNA was extracted using a Bacteria total RNA Isolation Kit (Sangon Biotech, Shanghai, China) and treated with RNase-free DNase (Takara, Dalian, China) to eliminate any residual DNA. The total RNA was added to the PCR system (used as temple) to check if the total DNA was completely hydrolyzed. This PCR system was used as a negative control. The cDNA was obtained using a PrimeScript RT reagent kit (TaKaRa, Dalian, China), and 2 μl of 1:5-diluted cDNA samples was used as the template for reverse transcription-PCR. The existence of the *cre* gene in cDNA was identified by RT-PCR. The 16S rRNA gene was used as a positive control. Primers used in RT-PCR are shown in Table 2.

**Table 2:** The primers used for PCR detection.

**Primers**	**Sequence (5′-3′)**	**PCR detection**
lox-F	GATGTGCTGCAAGGCGATTAAGTTG	Single-colony PCR
lox-R	GATCAGGTTGTGCAGCTGGTCAGCAG	
16s-RT-F	AAATTGAAGAGTTTGATCATGG	Reverse transcription PCR
16s-RT-R	TAAGGAGGTGATCCAACCGCAG	
Cre-RT-F	ATGTCCAATTTACTGACCGTACAC	
Cre-RT-R	CTAATCGCCATCTTCCAGCAG	
qGAP-F	ATCTTCCACTGGTGCTGCTA	Real-time quantitative PCR
qGAP-R	GGCATCTTCAGTGTAACCCA	
qAPPA-F	CTCAAAAGCAAGCCTACGG	
qAPPA-R	TGGAAGACCAACGAAACCT	

### Prevention of plasmid recombination and construction of pMCO-AOXα

To inhibit the leakage expression of Cre recombinase in *E. coli*, an upstream ORF or *lacO* (operator gene) was integrated using the primer pairs AOX-F/R-uORF or AOX-F/R-*lacO*, respectively. The resulting recombinant plasmids were named pMCU and pMCO. The sites of *Xho* I, *Not* I, and *Nco* I on pMCO were deleted by overlap extension PCR, making them available in the heterogenous expression cassette or linearization site of *HIS4*. The fragments AOX1TT and P_AOX1_-αFactor were subsequently integrated into pMCO. The resulting recombinant plasmids were named pMCO-AOXα.

### Construction of the tandem multiple copy *appA* expression vectors

The sequence the of *appA* gene according to the literature (Dassa et al., 1990) was codon optimized and synthesized *in vitro*, then cloned into the pMCO-AOXα vector via *Eco*R I and *Pst* I. The resulting recombinant plasmid, named pMCO-AOXα-A1, was digested by *Spe* I and *Xba* I to generate a 2.7 kb expression cassette. Meanwhile, pMCO-AOXα-A1 was also linearized by *Xba* I. The two fragments were connected by T4 ligase to construct the plasmid pMCO-AOXα-A2; the multicopy recombinant vectors pMCO-AOXα-A4 and pMCO-AOXα-A8 were constructed in the same way.

### Generation of recombinants with a multicopy *appA* gene and test tube-scale culture

The expression vectors pMCO-AOXα-An (*n* = 1, 2, 4, or 8) were linearized with *Sal* I and transformed into *P. pastoris* GS115 by electroporation. The charging voltage, resistance, and capacitance were 1,500 V, 250 Ω, and 25 μF, respectively. Positive transformants on YPDZ plates were shifted to new YPDZ plates with pre-marked serial numbers and incubated overnight at 30°C. The fresh clones were placed into 3 mL of BMGY liquid medium in test tubes and cultured at 30°C and 200 rpm for ~36 h until a stable saturation was attained; then, the cells were harvested by centrifugation and resuspended in 1 mL of BMMY. After incubation at 30°C and 200 rpm for 24 h, the enzyme assay was immediately performed.

### Phytase activity assay

Phytase activity was determined by the ferrous sulfate-molybdenum blue method (Huang et al., 2006). Briefly, 100 μl of 200-fold diluted enzyme solution was incubated with 900 μl of substrate solution (4 mM sodium phytate in 0.25 M sodium acetate buffer, pH 4.5) at 37°C for 10 min. The reaction was stopped by adding 1 mL of 10% (w/v) trichloroacetic acid (TCA). The amount of inorganic phosphate released was analyzed by adding 2 mL of a coloring reagent C [1% (w/v) ammonium molybdate, 3.2% (v/v) sulfuric-acid solution, and 7.2% (w/v) ferrous sulfate solution] and measuring the absorption at 700 nm. One unit (U) of phytase activity was defined as the amount of enzyme that releases 1 μmol phosphate in 1 min at 37°C.

### Determination of *appA* copy number by real-time quantitative PCR

In each group of recombinants with designed copy numbers (copy number = 1, 2, 4, or 8), the clone that exhibited the highest phytase activity was selected, and its genomic DNA was extracted using a Yeast Genomic DNA Isolation Kit (Sangon Biotech, Shanghai, China). GAPDH (PAS_chr2-1_0437), which is present as a single copy in the *P. pastoris* genome, was chosen as the reference gene. All primers used for real-time quantitative PCR (qPCR) are listed in Table 2. For each qPCR reaction mixture, 2 μl of 1:100 diluted genomic DNA samples was used as the template with 10 μl of SYBR Premix Ex Taq II (Takara, Dalian, China) in a total volume of 20 μl. The amplification reaction was performed according to the manufacturer's instructions. Each reaction was performed in triplicate. Relative quantification of the copy number of the target gene was performed according to the 2^−ΔΔCt^ method (Livak and Schmittgen, 2001; Zarei et al., 2014), where ΔΔ*C*t = Δ*C*t of target − Δ*C*t of calibrator, and Δ*C*t = *C*t of target of calibrator − *C*t of reference (GAPDH). A strain with a known copy number (one copy) of the target gene served as the calibrator strain.

### The obliteration of resistance toward zeocin for *P. pastoris* transformants

The 24-h BMMY cultures were plate streaked onto YPD plates and incubated at 30°C until colonies were visible. Single clones were shifted to new YPD and YPDZ plates and incubated at 30°C overnight. The percentage of cells that lost the Zeocin^R^ cassette after methanol induction was calculated by comparing colony numbers on YPD and YPDZ plates. Then, the phytase activity assay was performed as previously described to reconfirm the yield of phytase for colonies that were sensitive to Zeocin.

### Generation of the expression recombinants with 12 or 16 copies of *appA*

In the group of recombinants harboring 8 copies of the target gene, the clone that exhibited the highest phytase activity was induced in YPM liquid medium in test tube. The obliteration of resistance to Zeocin was performed as previously described. After reexamining the phytase activity of the clones that lost resistance to Zeocin, the linearized expression vectors pMCO-AOXα-A4 and pMCO-AOXα-A8 were transformed into markerless *P. pastoris* cells by electroporation, resulting in transformants with 12 or 16 copies of the target gene. The enzyme assay of positive transformants was determined in test tube-scale, and the copy number of the target gene was determined by qPCR as previously described.

### Shake-flask cultures

In each group of recombinants harboring 1, 2, 4, 8, 12, or 16 copies of the target gene, the clone that exhibited the highest phytase activity was cultured and induced on the shake-flask scale for reexamination. Transformants were transferred to 30 mL of BMGY liquid medium in a 250 mL shake flask and were cultured ~48 h at 30°C and 200 rpm until a stable saturation was attained. Then, the cells were harvested by centrifugation, resuspended in 30 mL of BMMY and cultured at 30°C at 200 rpm for 96 h. A total of 150 μl of 100% methanol was added every 24 h to maintain induction, and 150 μl of culture was extracted at the same time for the enzyme assay. After 96 h of induction, culture supernatants were analyzed by SDS-PAGE.

## Results

### Identification of leakage expression of cre regulated by AOX1 promoter in *E. coli*

**Figure 1:**
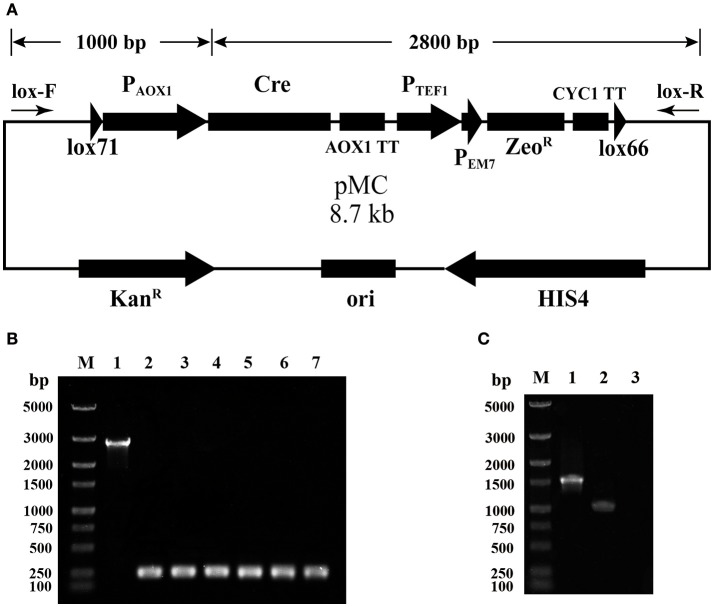
Identification of the leakage expression of Cre recombinase. **(A)** The map of plasmid pMC. The primer pairs lox-F/R are indicated by arrows, and the calculated molecular weight of the two detected fragments is shown on top. **(B)** Single-colony PCR analysis of *E. coli* (pMC). Line M, DNA marker; Line 1, the detected fragment of Cre-AOX1TT-P_TEF1_-P_EM7_-Zeo^R^-CYC1TT-*lox66*; Line 2–7, the detected fragment of *lox71*-P_AOX1_-Cre-AOX1TT-P_TEF1_-P_EM7_-Zeo^R^-CYC1TT-*lox66*. **(C)** RT-PCR analysis of *E. coli* (pMC). Line M, DNA marker; Line 1, positive control of 16s rRNA gene; Line 2, *cre* gene; Line 3, negative control.

A selection marker rescue vector pMC containing a *lox71*-Cre-Zeo^R^-*lox66* cassette was designated for multicopy *P. pastoris* expression strain generation. The schematic map of the construction of pMC is shown in Figure S1. Briefly, pUC57-MCS7 was used as the original plasmid, and four DNA fragments were subsequently integrated into pUC57-MCS7. To verify the proper assembly of pMC, single-colony PCR was performed using the primer pair lox-F/R, indicated by arrows in Figure 1A. The fragment of Cre-AOX1TT-P_TEF1_-P_EM7_-Zeo^R^-CYC1TT-*lox66* was ~2,800 bp, while the fragment plus *lox71*-P_AOX1_ was calculated as ~3,800 bp. Unexpectedly, a single DNA band of ~250 bp was clearly observed by gel electrophoresis analysis (Figure 1B), which indicated that the fragment of Cre-AOX1TT-P_TEF1_-P_EM7_-Zeo^R^-CYC1TT-*lox66* confirmed the estimated molecular weight (2.8 kb). The smaller electrophoretic band suggests that the plasmid pMC recombined spontaneously.

To further identify the leakage expression of the *cre* gene, the total RNA of *E. coli* DH10B (pMC) was extracted, and reverse transcription-PCR (RT-PCR) was performed. The negative control (Figure 1C, line 3) was not observed, demonstrating that the genome DNA and plasmid DNA were completely digested. The positive control (Figure 1C, line 1) and Cre recombinase (Figure 1C, line 2) were detected, indicating that the AOX1 promoter from *P. pastoris* could be partially recognized by *E. coli* and the downstream *cre* gene was transcribed. We deduced that the leakage expression of Cre recombinase caused the excision of *lox71*-P_AOX1_-Cre-AOX1TT-P_TEF1_-P_EM7_-Zeo^R^-CYC1TT-*lox66*, generating a *lox72* site on the plasmid.

### Blocking cre-mediated plasmid recombination with a *laco* operator

**Figure 2:**
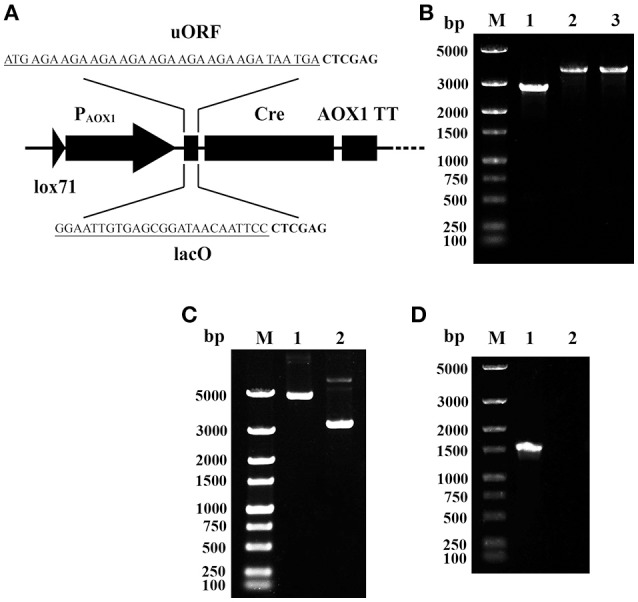
Prevention of Cre-mediated plasmid recombination. **(A)** Schematic map of the integration of uORF and *lacO*. The sequences of uORF and *lacO* are underlined; the downstream *Xho* I sites are in bold. **(B)** Single-colony PCR analysis. Line M, DNA marker; Line 1, the detected fragment of Cre-AOX1TT-P_TEF1_-P_EM7_-Zeo^R^-CYC1TT-*lox66*; Line 2, the detected fragment between *lox71* and *lox66* of pMCO; Line 3, the detected fragment between *lox71* and *lox66* of pMCU. **(C)** Gel electrophoresis analysis of extracted recombinant plasmids from *E. coli*. Line M, DNA marker; Line 1, pMCO; Line 2, pMCU. **(D)** RT-PCR analysis of *E. coli* (pMCO). Line M, DNA marker; Line 1, positive control of 16s rRNA gene; Line 2, *cre* gene.

To overcome the leakage expression of Cre in *E. coli*, two cis elements were integrated between P_AOX1_ and the *cre* gene on the plasmid pMC (shown in Figure 2A). One is an upstream ORF (uORF), which contains eight AGA codons. The other is the lac operator gene (*lacO*) of the lactose operon. The integrations were performed using primer pairs AOX-F/AOX-R-uORF and AOX-F/AOX-R-*lacO*, respectively. The resulting recombinant plasmids were named pMCU and pMCO.

To verify the proper assembly of pMCU and pMCO, colony PCR was performed using the primer pair lox-F/R. A 3.8 kb DNA band was observed for both plasmids (Figure 2B, lane 2–3), which matches the predicted size of the whole insertion fragment, indicating the proper integration of P_AOX1_-uORF and P_AOX1_-*lacO*. However, when these two recombinant plasmids were extracted, gel electrophoresis analysis showed that the size of the two plasmids was clearly different. The larger band (Figure 2C, lane 1, consistent with the theoretical molecular weight) showed the outstanding stability of pMCO, while the smaller band of pMCU (Figure 2C, lane 2) indicated that recombination between *lox71* and *lox66* mediated by Cre recombinase still occurred. The stability of pMCO was also verified by RT-PCR using the primer pairs 16s-RT-F/R and Cre-RT-F/R. The gel electrophoresis analysis showed that no cDNA of the *cre* gene was detected (Figure 2D, lane 2). These results showed that the *lacO* operator could effectively block the leakage expression of Cre recombinase promoted by the AOX1 promoter in *E. coli*.

### Construction of the expression vector pMCO-AOXα for multiple integration

**Figure 3:**
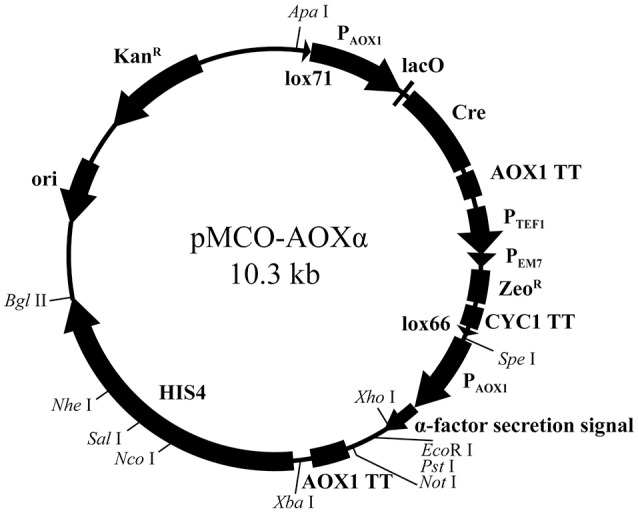
Scheme of the expression vector pMCO- AOXα.

pMCO was applied to construct the full functional expression vector for *P. pastoris*. First, to make *Xho* I, *Not* I, and *Nco* I available in the heterogenous expression cassette or linearization site of *HIS4*, these sites on pMCO were deleted by overlap extension PCR. Then, the fragments AOX1TT and P_AOX1_-αFactor were subsequently integrated into pMCO. The resulting recombinant plasmid was named pMCO-AOXα (Figure 3).

The expression vector pMCO-AOXα has the potential for commercial application: it can be linearized by *Nco* I, *Sal* I, and *Nhe* I on *HIS4*, and heterologous genes can be integrated into the expression cassette by utilizing the *Xho* I, *Eco*R I, *Pst* I, and *Not* I sites. Since *Spe* I and *Xba* I were introduced into pMCO-AOXα as isocaudamers, the expression cassette can be inserted in the unique *Xba* I site repetitively, generating a multicopy recombinant plasmid. Briefly speaking, suitable restriction endonuclease sites facilitate the insertion of target genes, the multimerization of expression cassettes and the integration of linearized recombinant plasmids.

### Construction of the tandem multiple copy *appA* expression vectors

**Figure 4:**
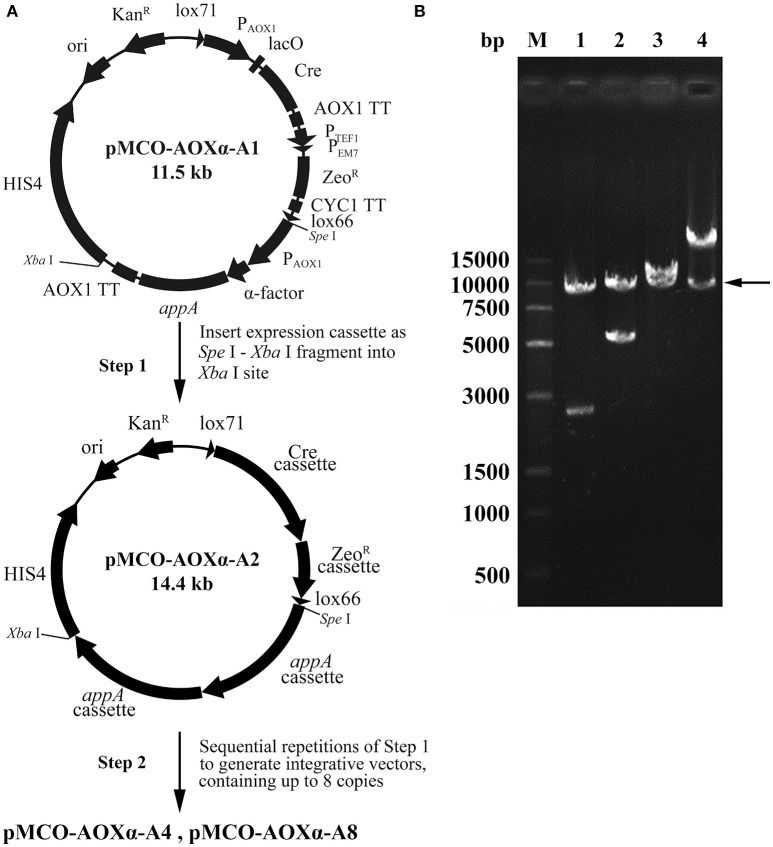
Scheme for construction and identification of the expression vectors with 1, 2, 4, or 8 copies of the *appA* gene. **(A)** Schematic map of the construction of expression vectors pMCO-AOXα-A1, pMCO-AOXα-A2, pMCO-AOXα-A4, and pMCO-AOXα-A8. **(B)** Gel electrophoresis analysis of recombinant vectors digested with *Spe* I and *Xba* I. Line M, DNA marker; Line 1, pMCO-AOXα-A1; Line 2, pMCO-AOXα-A2; Line 3, pMCO-AOXα-A4; Line 4, pMCO-AOXα-A8.

Phytase is generally used as an animal feed supplement to enhance the nutritive value of plant material by liberation of inorganic phosphate from phytic acid. Phytase AppA was from *E. coli* and can catalyze the hydrolysis of phytic acid (myo-inositol hexakisphosphate). The optimized *appA* gene was synthesized and successfully cloned into pMCO-AOXα; the resulting vector carrying 1 copy of the *appA* gene was designated as pMCO-AOXα-A1. The multimerization was performed as described in the “Materials and methods,” producing the vectors pMCO-AOXα-A2, pMCO-AOXα-A4 and pMCO-AOXα-A8, which carried 2, 4, or 8 copies of the *appA* gene, respectively (Figure 4A). To confirm the correct orientation of the integration of expression cassette, which was inserted in the unique *Xba* I site, the recombinant vectors pMCO-AOXα-A1, pMCO-AOXα-A2, pMCO-AOXα-A4, and pMCO-AOXα-A8 were digested with *Spe* I and *Xba* I (Figure 4B). Gel electrophoresis analysis showed that the molecular weight of the inserted expression cassettes increased gradually, while the molecular weight of the vector frame remained ~10 kb (arrow, Figure 4B). The result verified that pMCO-AOXα-A1, pMCO-AOXα-A2, pMCO-AOXα-A4, and pMCO-AOXα-A8 were constructed correctly.

### Construction of transformants with 1, 2, 4, or 8 copies of the *appA* gene

The plasmids containing 1, 2, 4, or 8 copies of *appA* were linearized and electroporated into *P. pastoris*. After selection of recombinants on YPDZ plates, 10 positive clones for GS115-pMCO-AOXα-An (*n* = 1, 2, 4, or 8) were picked (a total of 40 clones), initially cultured in BMGY, then transferred into BMMY to induce the expression of the target protein with methanol. The supernatants of 24-h BMMY cultures were collected, and the highest-yielding clones were screened by quantification of phytase activity.

**Figure 5:**
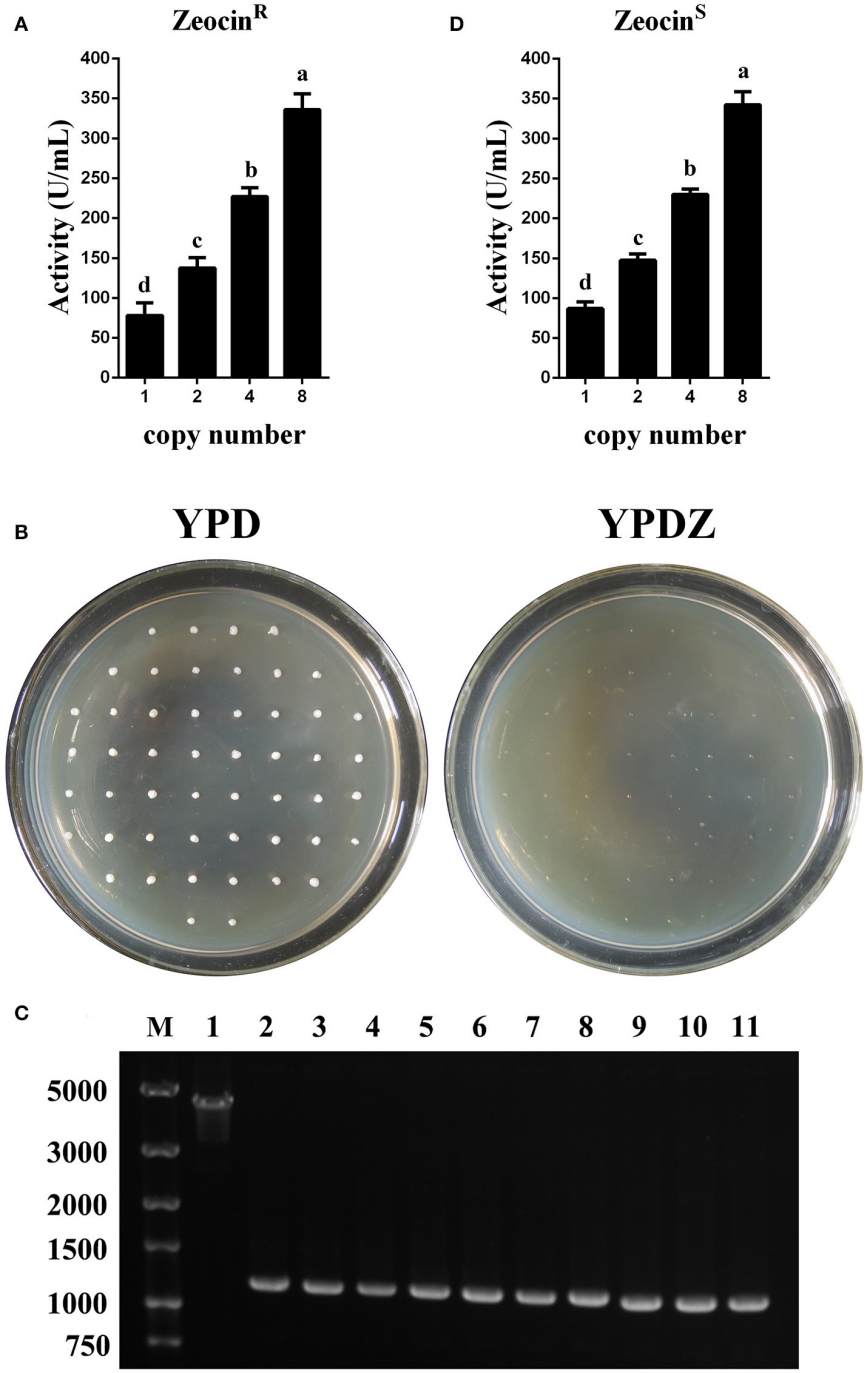
Identification and expression analysis of the transformants that were resistant or sensitive to Zeocin. **(A)** Expression analysis of the Zeocin^R^ transformants. **(B)** Identification of the colonies that were sensitive toward Zeocin. **(C)** Single-colony PCR analysis of Zeocin^S^ strains using primer pairs lox-F/αf-R. Line M, DNA marker; Line 1, Zeocin^R^ transformants; Line 2-11, 10 randomly selected Zeocin^S^ transformants. **(D)** Expression analysis of the Zeocin^S^ transformants. The experiments were performed 10 or 20 times as described; the mean values ± *SD* are presented. Bars with the same letters are not significant (*P* < 0.05).

In test tube culture, the transformants GS115-pMCO-AOXα-An-Zeocin^R^ (*n* = 1, 2, 4, or 8) showed an increasing trend in the recombinant protein expression level, and the GS115-pMCO-AOXα-A8-Zeocin^R^ obtained the highest phytase yield (Figure 5A). For each group of transformants GS115-pMCO-AOXα-An-Zeocin^R^ (*n* = 1, 2, 4, or 8), the production of the strain exhibiting the highest yield of phytase from the 10 clones is listed in Table 3.

**Table 3:** The phytase yield of the Zeocin^R^ transformants at the test tube scale.

**Strains**	**Exhibited highest yield of phytase (U/mL)**	**Improvement of yield of recombinant protein (fold)**
GS115-pMCO-AOXα-A1-Zeocin^R^ #1	93.97	1.00
GS115-pMCO-AOXα-A2-Zeocin^R^ #7	150.61	1.60
GS115-pMCO-AOXα-A4-Zeocin^R^ #5	238.22	2.54
GS115-pMCO-AOXα-A8-Zeocin^R^ #3	355.82	3.79
GS115-pMCO-AOXα-A8A4-Zeocin^R^ #7	418.23	4.45
GS115-pMCO-AOXα-A8A8-Zeocin^R^ #9	369.88	3.94

Since methanol could also induce the expression of Cre recombinase, the loss of Zeocin resistance gene was checked. The BMMY cultures of the clones denoted as GS115-pMCO-AOXα-A1-Zeocin^R^ #1, GS115-pMCO-AOXα-A2-Zeocin^R^ #7, GS115-pMCO-AOXα-A4-Zeocin^R^ #5, and GS115-pMCO-AOXα-A8-Zeocin^R^ #3 were streaked onto YPD plates, single colonies were transferred to new YPD and YPDZ plates, and the percentage of cells that lost the Zeocin^R^ cassette was calculated (Figure 5B, 10 colonies for each Zeocin^R^ transformant except 20 colonies for GS115-pMCO-AOXα-A8-Zeocin^R^ #7). All clones were sensitive to Zeocin, indicating the excision of the resistance gene by Cre recombinase, which was verified by single-colony PCR (Figure 5C, and the schematic map of the deletion of Zeocin resistance gene was shown in Figure S2). Then, the phytase yield assay was performed for these 50 colonies (Figure 5D, in test tube-scale as well); the strain exhibiting the highest yield of phytase from each group of 10 or 20 clones is listed in Table 4. The yield of phytase and the copy number analysis are also shown in Table 4.

**Table 4:** The phytase yield and the copy number analysis of the Zeocin^S^ transformants.

**Strains**	**Exhibited highest yield of phytase (U/mL)**	**Improvement of yield of recombinant protein (fold)**	**Copy number of *appA*^a^**
GS115-pMCO-AOXα-A1-Zeocin^S^ #2	95.11	1.00	1.00
GS115-pMCO-AOXα-A2-Zeocin^S^ #3	153.43	1.61	1.82 ± 0.06
GS115-pMCO-AOXα-A4-Zeocin^S^ #1	240.73	2.53	3.49 ± 0.13
GS115-pMCO-AOXα-A8-Zeocin^S^ #17	358.38	3.77	7.34 ± 0.65
GS115-pMCO-AOXα-A8A4-Zeocin^S^ #5	417.79	4.39	11.08 ± 0.51
GS115-pMCO-AOXα-A8A8-Zeocin^S^ #7	371.52	3.91	14.75 ± 0.29

a *The copy number of appA in the calibrator strain harboring plasmid pMCO-AOXα-A1 was defined as 1 copy*.

### Repetitive transformation to construct transformants with 12 or 16 copies of *appA*

**Figure 6:**
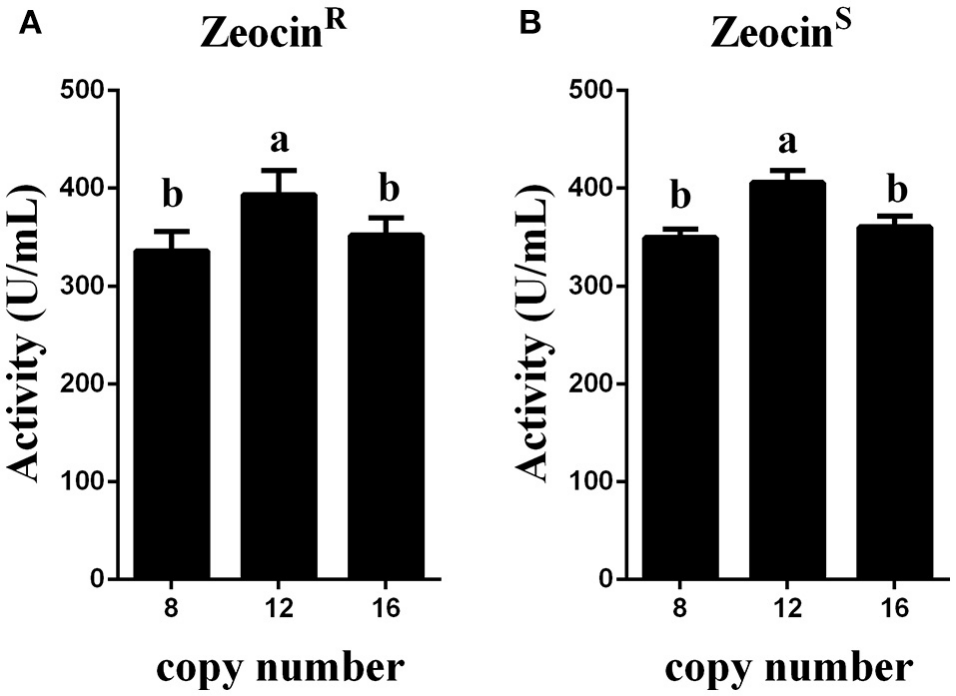
Expression analysis of the transformants that were resistant or sensitive to Zeocin. **(A)** Expression analysis of the Zeocin^R^ transformants. **(B)** Expression analysis of the Zeocin^S^ transformants. The experiments were performed 10 times as described; the mean values ± *SD* are presented. Bars with the same letters are not significant (*P* < 0.05).

To obtain transformants with 12 or 16 copies of the *appA* gene, the vectors pMCO-AOXα-A4 or pMCO-AOXα-A8 were successfully transformed into GS115-pMCO-AOXα-A8-Zeocin^S^ #17, and the resulting strains were denoted as GS115-pMCO-AOXα-A8A4-Zeocin^R^ or GS115-pMCO-AOXα-A8A8-Zeocin^R^, respectively. The results showed that our marker rescue system worked well in *P. pastoris*. For each strain, 10 positive clones were cultured and induced; their phytase expression levels are shown in Figure 6A. In addition, the highest-yielding clones for GS115-pMCO-AOXα-A8A4-Zeocin^R^ and GS115-pMCO-AOXα-A8A8-Zeocin^R^ were screened (listed in Table 3). The highest phytase yield of the transformants harboring 12 copies of *appA* gene was 418.23 U/mL, which was 4.45 and 1.17 times higher than the yield of 1-copy transformants (93.97 U/mL) and 8-copy transformants (355.82 U/mL), respectively. However, the phytase yield of 16-copy transformants decreased to 369.88 U/mL, indicating that the abundant over-expression of proteins imposed a metabolic burden on the secretory pathway.

Zeocin resistant genes of the *P. pasrois* transformants #7(12 copies) and #9(16 copies) were further deleted, the phytase yields of the 10 Zeocin^S^ colonies were assayed (as shown in Figure 6B). The strains exhibiting the highest phytase yield from each group of 10 clones of GS115-pMCO-AOXα-A8A4-Zeocin^S^ or GS115-pMCO-AOXα-A8A8-Zeocin^S^ are listed in Table 4. The yield of phytase and the copy number analysis are also shown in Table 4.

### Shake-flask cultures

**Figure 7:**
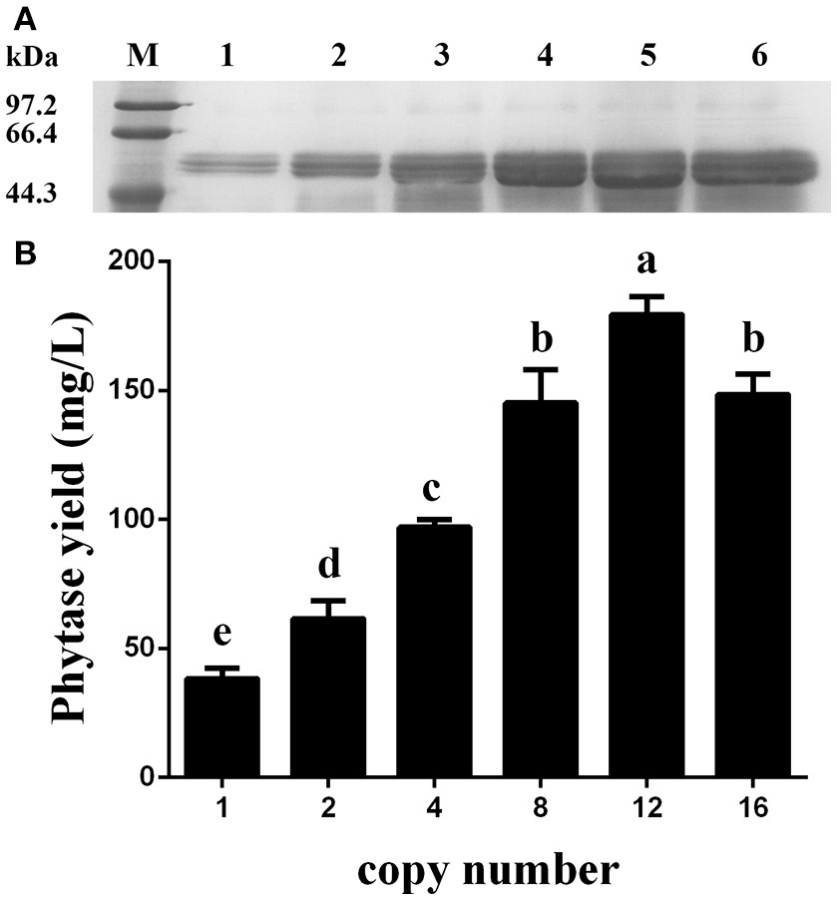
Expression analysis of the Zeocin^S^ transformants. **(A)** SDS-PAGE analysis. Line M: protein marker; Line 1: GS115-pMCO-AOXα-A1-Zeocin^S^ #2; Line 2: GS115-pMCO-AOXα-A2-Zeocin^S^ #3; Line 3: GS115-pMCO-AOXα-A4-Zeocin^S^ #1; Line 4: GS115-pMCO-AOXα-A8-Zeocin^S^ #17; Line 5: GS115-pMCO-AOXα-A8A4-Zeocin^S^ #5; Line 6: GS115-pMCO-AOXα-A8A8-Zeocin^S^ #7. **(B)** Production of phytase with different copy number of the *appA* gene. The experiments were performed 3 times; the mean values ± *SD* are presented. Bars with the same letters are not significant (*P* < 0.05).

After the generation and identification of a series of variant *P. pastoris* transformants harboring 1, 2, 4, 8, 12, or 16 copies of the *appA* gene, the shake-flask cultures were created to reconfirm the phytase yield for each transformant. Data shown in Figure 7 demonstrated that the recombinant phytase yield increased as the copy number of *appA* gene increased, and GS115-pMCO-AOXα-A8A4-Zeocin^S^ #5 reached the highest expression level of 179.34 mg/L, based on specific activity of 3,100 U/mg protein (Huang et al., 2006). The phytase yield of GS115-pMCO-AOXα-A8A8-Zeocin^S^ #7 decreased to 148.38 mg/L, probably because of an ERAD response to restore ER homeostasis.

## Discussion

*P. pastoris* has been widely used for the industrial production of recombination proteins because of its efficient protein secretion and high cell density cultivation. Several studies have reported that the gene copy number has a significant impact on heterogenous protein production, which is useful to achieve a high expression level of a target protein (Damasceno et al., 2012; Ahmad et al., 2014). PTVA method is the most commonly used protocol to generate multiple copy strains. High copy number strains were selected under high Zeocin concentration randomly (Aw and Polizzi, 2013). Rational design of overexpression strain with certain copy number of the target gene is difficult since the limited selection pressure offered by the host (Zhu et al., 2009). Therefore, the hypothesis that the deletion of marker genes in *P. pastoris* facilitates the repetitive integration of recombinant vector was investigated.

Two marker rescue (deletion) systems were compared, and the leakage expression of Cre recombinase in *E. coli* was examined. In the first marker rescue system (self-unexcisable method), the *loxP-*Marker*-loxP* fragment and the plasmid carrying Cre recombinase were transformed into *P. pastoris* successively (Gueldener et al., 2002). In the second marker rescue system (self-excisable method), the PCR fused *lox71*-Cre-Zeo^R^-*lox66* fragment was transformed into *P. pastoris* by electroporation. Methanol then induced the expression of Cre recombinase, which excises the fragment of *lox71*-Cre-Zeo^R^-*lox66*, leaving a *lox72* site in the *P. pastoris* genome (Pan et al., 2011). The self-excisable marker recycling system has the advantage of direct and easy operation, but still has the bottleneck of leakage expression of Cre recombinase, identified by single-colony PCR (Figure 1B) and RT-PCR (Figure 1C). The mechanism for spontaneous plasmid recombination may be that *E. coli* can recognize the AOX1 promoter and initiate the expression of Cre recombinase. Although the transcription level of Cre recombinase might be very low, the trace amount of recombinase could still cause recombination between *lox71* and *lox66*.

The introduction of an intron in the *cre* gene has been shown to effectively block the leakage expression of Cre recombinase in *E. coli* because *E. coli* lacks the ability to process introns in mRNA (Agaphonov and Alexandrov, 2014). The introduced intron is from the *COF1* gene of *H. polymorpha*. Once the intron is replaced by another eukaryotic intron (such as the intron of the *xynG2* gene from *Aspergillus oryzae*), this method can be easily adapted for other fungal species (Zhang et al., 2016). In our work, *lacO* was inserted between P_AOX1_ and the *cre* gene. The operator gene *lacO* was bound with repressor protein LacI in *E. coli*; therefore, the downstream gene expression was blocked. In *P. pastoris, lacO* cannot be recognized because of the absence of LacI, so the downstream *cre* gene can be induced normally. This was demonstrated by both single-colony PCR and RT-PCR (Figure 2). Compared with the intron method, the *lacO* method can work in any fungal cell that does not harbor the repressor protein LacI, theoretically. The *lacO* method averts the screening of the intron sequences that are indispensable to be genetically manipulated, which might be laborious and time-consuming. The recombinant vector pMCO-AOXα containing the complete expression cassette for a heterogenous gene could be universally employed in the construction of a multicopy integration strain in *P. pastoris* by rescuing the selection marker with Cre combinase and has the potential for commercial application.

In this study, a series of *P. pastoris* transformants with 1, 2, 4, 8, 12, or 16 copies of the *appA* gene were generated. The 1-, 2-, 4-, and 8-copy transformants were constructed by transformation of pMCO-AOXα-An (*n* = 1, 2, 4, or 8) into GS115, followed by deleting the Cre-Zeo^R^ cassette. The 12- and 16-copy transformants were constructed by transformation of pMCO-AOXα-An (*n* = 4 or 8) into GS115-pMCO-AOXα-A8-Zeocin^S^ #17; the resulting Zeocin^R^ clones were also treated with methanol to generate a Zeocin^S^ strain. The Zeocin^S^ strain can easily absorb other linearized recombinant vectors and integrate them into its genome. The qPCR analysis and the phytase yield assay showed a positive correlation between gene copy number and the recombinant protein expression level; GS115-pMCO-AOXα-A8A4-Zeocin^S^ obtained the highest phytase yield (shown in Table 4, Figure 7). However, the phytase yield of 16-copy transformants decreased. We deduced that abundantly over-expressed protein might remain unfolded and activate the unfolded protein response (UPR) leading to endoplasmic reticulum-associated degradation (ERAD) (Malhotra and Kaufman, 2007; Vembar and Brodsky, 2008; Aw and Polizzi, 2013; Puxbaum et al., 2015).

The promoter region of *Spe* I-P_AOX1_-αfactor-*Eco*R I and the recombination site *Xba* I-*HIS4*-*Bgl* II of pMCO-AOXα can be easily changed and optimized. For instance, the recently discovered promoters such as G6 (Prielhofer et al., 2013), GCW14 (Liang et al., 2013), and THI11 (Stadlmayr et al., 2010), with their own characteristics, can be inserted into pMCO easily via *Spe* I and *EcoR* I sites. Another case is that the *HIS4* gene can be replaced with other DNA fragments such as non-transcribed intergenic spacer (NTS) of the rDNA repeat locus (Marx et al., 2009), or other genes denoted as “non-essential protein of unknown function” (PAS_CHR1-1_0141, PAS_CHR1-3_0180, PAS_CHR1-3_0216 and PAS_CHR4_0177). Multiple integration of vectors into *P. pastoris* in a head-to-tail orientation can raise the possibility of excision of the homologous region (loop out region) (Aw and Polizzi, 2013). pMCO-AOXα can be used in the dispersed integration of the target gene by substituting the *HIS4* recombination site with various homologous recombination fragments to reduce the potential genetic instability of the recombinant strain in *P. pastoris*.

## Author contributions

DL performed all the experiments, coordinated the data analysis, and prepared the manuscript. BZ, SL, and JZ contributed in the preparation of the manuscript and data analysis. HC and YH provided the research work suggestion. ZC supervised the whole study.

### Conflict of interest statement

The authors declare that the research was conducted in the absence of any commercial or financial relationships that could be construed as a potential conflict of interest. The reviewer XZ and handling Editor declared their shared affiliation.

## Supplementary material

Figure S1 Schematic map of the construction of expression vector pMC.

Figure S2 Schematic map of the excision of the resistance gene.
