# Supplementary figures and images for: A Novel Vector for Construction of Markerless Multicopy Overexpression Transformants in Pichia pastoris
Source: Front Microbiol. 2017 Sep 11;8:1698. doi: 10.3389/fmicb.2017.01698 (PMC5601908; doi:10.3389/fmicb.2017.01698)

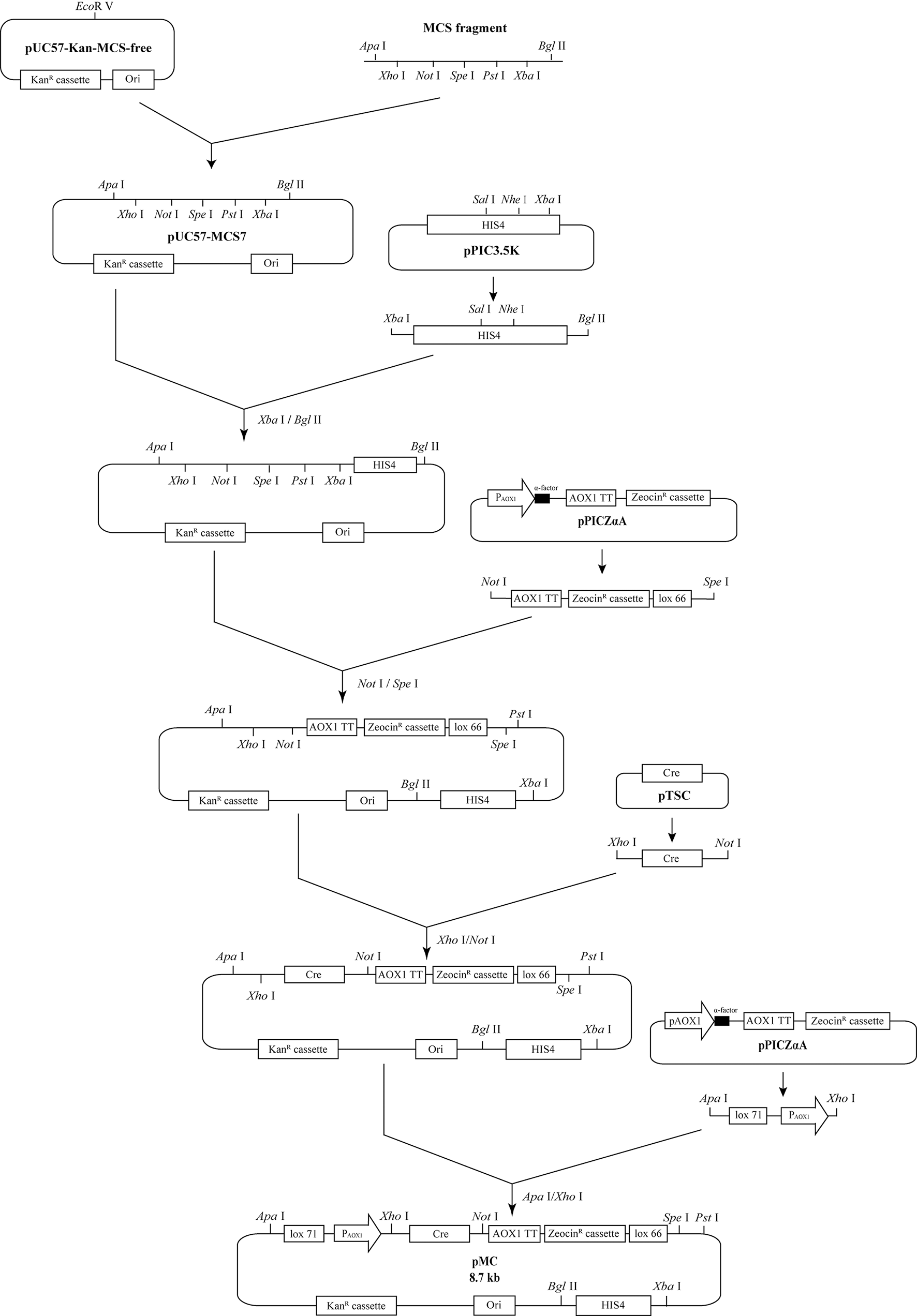

Supplement: Figure S1 — Schematic map of the construction of expression vector pMC. [file Image1.TIF]

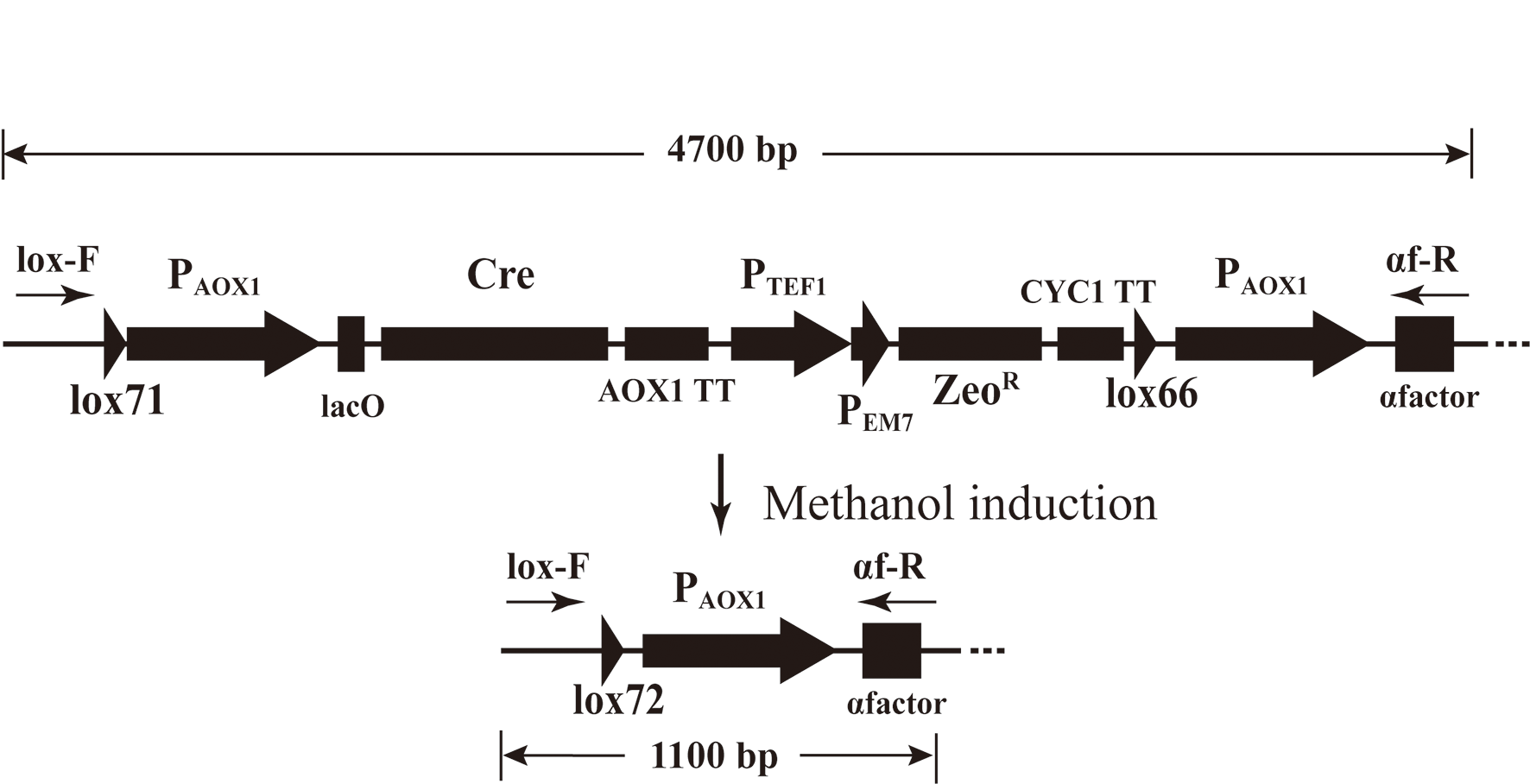

Supplement: Figure S2 — Schematic map of the excision of the resistance gene. [file Image2.TIF]
